# Improving physical health and reducing substance use in psychosis – randomised control trial (IMPACT RCT): study protocol for a cluster randomised controlled trial

**DOI:** 10.1186/1471-244X-13-263

**Published:** 2013-10-16

**Authors:** Fiona Gaughran, Daniel Stahl, Khalida Ismail, Zerrin Atakan, John Lally, Poonam Gardner-Sood, Anita Patel, Anthony David, David Hopkins, Bee Harries, Philippa Lowe, Diana Orr, Maurice Arbuthnot, Robin M Murray, Kathryn E Greenwood, Shubulade Smith

**Affiliations:** 1 National Psychosis Service, South London and Maudsley NHS Foundation Trust, Denmark Hill, London, UK; 2 Institute of Psychiatry and the Biomedical Research Centre, BRC Nucleus, Maudsley Hospital, South London and Maudsley NHS Foundation Trust, Denmark Hill, London, UK; 3 Department of Biostatistics, Institute of Psychiatry, King’s College London, London, UK; 4 Institute of Psychiatry, King’s College London, London, UK; 5 King’s College Hospital NHS Foundation Trust, London, UK; 6 Section of Neuroimaging, Department of Psychosis Studies, Institute of Psychiatry, King’s College London, London, UK; 7 Department of Psychosis Studies, Institute of Psychiatry, King’s College London, London, UK; 8 Centre for the Economics of Mental and Physical Health (CEMPH), Institute of Psychiatry at King's College London, London, UK; 9 Division of Ambulatory Care & Local Networks, King’s College Hospital NHS Foundation Trust, London, UK; 10 King’s College London School of Medicine, London, UK; 11 Department of Mental Health Sciences, Royal Free and University College Medical School, London, UK; 12 School of Psychology, University of Sussex, Brighton, UK; 13 Early Intervention in Psychosis Service, Sussex Partnership NHS Foundation Trust, Worthing, West Sussex, UK; 14 South London and Maudsley NHS Foundation Trust, London, UK

**Keywords:** Severe mental illness, Schizophrenia, Psychosis, Metabolic syndrome, Health promotion, Randomised controlled trial

## Abstract

**Background:**

Cardiovascular morbidity and mortality is increased in individuals with severe mental illnesses.

We set out to establish a multicentre, two arm, parallel cluster randomized controlled trial (RCT) of a health promotion intervention (HPI), IMPACT Therapy. The patient-tailored IMPACT Therapy aims to target one or more health behaviours from a pre-defined list that includes cannabis use; alcohol use; other substance use; cigarette smoking; exercise; diet and diabetic control, prioritising those identified as problematic by the patient, taking a motivational interviewing and CBT approach.

**Methods:**

Impact therapy will be delivered by care coordinators in the community to the treatment group and will be compared to treatment as usual (TAU). The main hypothesis is that the addition of IMPACT Therapy (HPI) to TAU will be more effective than TAU alone in improving patients’ quality of life as measured by the Short Form-36, including mental health and physical health subscales on completion of the intervention at 12 months post randomisation. A subsidiary hypothesis will be that addition of IMPACT Therapy (HPI) will be more cost-effective than TAU alone in improving health in people with SMI 12 months from baseline. The IMPACT therapy patient groups’ improvement in quality of life, as well as its cost effectiveness, is hypothesised to be maintained at 15 months. Outcomes will be analyzed on an intention-to-treat (ITT) basis.

**Discussion:**

The results of the trial will provide information about the effectiveness of the IMPACT therapy programme in supporting community mental health teams to address physical comorbidity in severe mental illness.

**Trial registration:**

ISRCTN58667926.

## Background

People with severe mental illnesses (SMI) such as schizophrenia, schizoaffective disorder and bipolar affective disorder die up to 20–25 years earlier than the general population[1-4]. Most of this premature mortality is due to physical disorders [5,6] with cardiovasular disease [7-9] and specially prominent. Worryingly, the mortality gap between those with schizophrenia and the general population appears to have widened in recent years [10].

High rates of modifiable cardiovascular disease (CVD) risk factors are seen in SMI, [11,12] including abdominal obesity; insulin resistance/glucose intolerance; hypertension; and dyslipidaemia;[13] these factors increase the chance of developing complications such as diabetes, heart disease, stroke, amputation, renal failure, blindness and ultimately, early death [14-17]. Antipsychotic medications accelerate weight gain and the onset of diabetes.[18-21]Once CVD risk factors develop, impaired motivation in SMI makes implementing lifestyle change challenging, [22,23] again increasing the risk of complications. On top of that, episodes of acute psychosis may interrupt diabetic control.

It is possible to prevent the development of CVD risk factors in SMI [24] and strategies exist to manage existing CVD risk [25]. The CATIE study confirmed that smoking cessation, nutrition counselling and supervised exercise programmes can help to reduce cardiovascular mortality in SMI [26], but this can be difficult to institute in practice. The UK government now emphasises the importance of health promotion in reducing cardiovascular disease burden in SMI [27,28]. However,no health promotion programmes have yet been adequately demonstrated in the UK to be reliable, reproducible and workable across the National Health Service (NHS).

To complicate matters, more people with SMI smoke cigarettes, [29,30] and use cannabis and other illicit drugs than do the general population [31,32]. On-going cannabis use leads both to poorer mental and physical health outcomes [33-37] with cannabis users on psychiatric intensive care units having higher serum glucose levels and heavier weights compared to non-cannabis users [38]. More recently developed treatment programmes for people with both psychosis and substance use show some promise [39-41] but are lengthy, complex and expensive. In clinical practice of course, parallel attendance at separate treatment programmes for physical health and drug use is often impractical.

A more practical alternative to separately addressing physical health and substance use may be an intervention targeting both lifestyle and substance use to maximize physical and mental health. We have developed a modular, manualised, health promotion intervention, IMPACT therapy [42], covering physical health, mental health and substance use, integrating the combined benefits of motivational interviewing and cognitive behavioural therapy (CBT) approaches at both individual and group level to effect lifestyle change. [42] This paper describes the study protocol for the Improving physical health and reducing substance use in Psychosis (IMPACT) randomised controlled trial (IMPACT RCT) to assess the effectiveness of IMPACT therapy (i.e. health promotion intervention (HPI)).

### Objectives

The primary objective is to determine the effectiveness and cost-effectiveness of the addition of an intensive health promotion intervention (IMPACT Therapy), designed to improve physical health and reduce substance use, to usual mental health care delivered by care coordinators (TAU) in people with severe mental illness (SMI) (defined as schizophrenia (ICD-10 code: F20), delusional Disorder (F22.0), schizoaffective disorder (F25), bipolar affective disorder (F31), recurrent depressive disorder (F32), current episode severe with psychotic symptoms (F33.3)).

The main hypothesis is that the addition of IMPACT Therapy (HPI) to TAU will be more effective than TAU alone in improving patients’ quality of life as measured by the Short Form-36, including mental health and physical health subscales [43] on completion of the intervention at 12 months post randomisation. This 12 month period incorporates an initial 3 months post randomisation to train the care coordinators to provide the IMPACT Therapy (HPI) over the following 9 months. We also hypothesise that the effect will be sustained three months after the end of the intervention, at 15 month follow up.

Our secondary hypothesis is that the addition of IMPACT Therapy (HPI) will be more cost-effective than TAU alone in improving health in people with SMI 12 months from baseline and that this cost-effectiveness will be sustained 3 months later at 15-month follow-up.

Subsidiary Hypotheses include

1. TAU plus IMPACT Therapy will reduce waist circumference by at least 1 cm at one year, compared to TAU alone

2. TAU plus IMPACT Therapy will be more effective in reducing weight at one year, compared to TAU alone

3. TAU plus IMPACT Therapy will result in a 50% reduction in proportion of people using cannabis compared to TAU alone.

4. TAU plus IMPACT Therapy will be more effective in reducing symptoms of psychosis compared to TAU alone

## Methods

### Design

**Figure 1:**
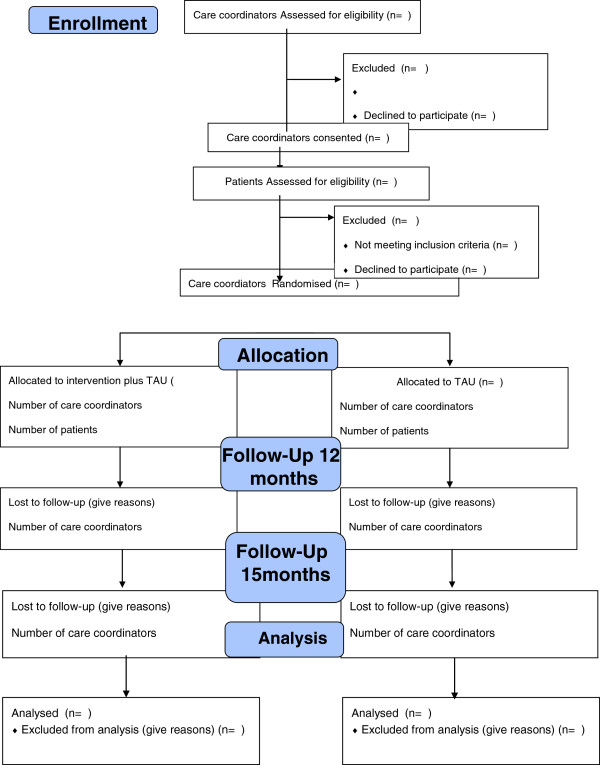
Summary of Trial design for Improving Physical health and reducing substance use in Psychosis – Randomised Control Trial (IMPACT RCT): Study protocol.

A multicentre, two arm, parallel cluster randomized controlled trial (RCT) of an health promotion intervention, (HPI) IMPACT Therapy. The study was planned and implemented in concordance with the Consolidated Standards of Reporting Trials (CONSORT) cluster trial extension standards [44,45] to compare the cost-effectiveness of combining treatment as usual (TAU) plus IMPACT Therapy, versus TAU alone in improving health at one-year follow-up. Figure 1 summarises the trial design.

### Setting

The study will take place within community mental health teams (CMHTs), including continuing care/recovery teams; community rehabilitation; assertive outreach and community forensic teams in six Mental Health NHS Trusts in the urban and rural locations of South London, Sussex, Somerset, Staffordshire and Shropshire. We will not recruit from specialist first episode psychosis teams.

### Participants

Care coordinators permanently employed and intending to remain so for a period of one year within the CMHT settings above, with a minimum of four patients on their caseload with a primary diagnosis of SMI, are eligible for recruitment to the trial.

Patients of participating care co-ordinators are eligible for inclusion in the study if:

a) Aged between 18–65 years old.

b) A diagnosis of a psychotic disorder (ICD 10 diagnosis F20-29, F31.2, F31.5,)

Exclusion criteria:

a) A primary diagnosis of learning disability.

b) A co-existing physical health problem that would, in the opinion of the medical investigators, independently impact on metabolic measures and/or substance use habits.

c) Current pregnancy, plus mothers less than 6 months post-partum.

d) Life threatening or terminal medical conditions where intensive care is already provided.

### Pre-randomisation

All care-coordinators in participating CMHTs will be offered best practice treatment as usual training on physical health awareness, including the provision of health promotion leaflets on healthy dietary routines and physical exercise, together with information on general and community support for a healthy lifestyle. The purpose of this is to ensure that all care-coordinators, irrespective of which treatment arm they are allocated to, have the same baseline level of understanding of physical health issues, thus ensuring more standardised treatment as usual.

### Selection and randomisation

This is a cluster randomised trial and the randomisation will take place at the level of the care co-ordinator. To select the order in which first care co-ordinators and then patients are approached, a random numbers generator will be used. Researchers will approach each eligible care coordinator in this order and seek consent to participate in the trial, working down the list until the target sample size is achieved.

Prior to randomisation, consenting care-coordinators will provide a list of their current patient caseload. Patients on that caseload meeting the inclusion criteria will be entered into the random numbers generator to create a randomly ordered list in which to approach potential participants. Researchers will then approach these patients sequentially and seek informed consent to participate in the RCT. In situations where a patient does not wish to take part in the study, the researcher will select another patient from the list in that random order.

After completing baseline assessments on all consenting patients in a care co-ordinator’s caseload, the clinical trials unit will conduct randomisation of the care coordinators, stratified by borough, using randomisation blocks of random sizes to either treatment arm (IMPACT Therapy) or treatment as usual (TAU).

Researchers and the statistician will remain blind to treatment allocation. Outcome assessments will only be conducted by researchers blind to the treatment arm. Any violations of the study protocol will be recorded and reported to the Trial Steering Committee and the Data Monitoring and Ethics Committee.

### Intervention: IMPACT therapy

All consenting community care coordinators randomised to the IMPACT Therapy arm will receive 4-day IMPACT training on physical health and substance use in SMI, as well as training in motivational interviewing (MI), cognitive behavioural therapy (CBT) techniques and in running groups to deliver health promotion. The patient-tailored IMPACT Therapy aims to target one or more health behaviours from a pre-defined list that includes cannabis use; alcohol use; other substance use; cigarette smoking; exercise; diet and diabetic control, prioritising those identified as problematic by the patient, taking a MI and CBT approach. Participants may start the community-based IMPACT Therapy as soon as they are well enough to attend, even if they are in-patients, to mirror clinical practice. IMPACT Therapy is supported by a manual, a reference book and a service user handbook [46]. Each care coordinator in the active intervention arm of the study will receive copies of these books.

Care Coordinators allocated to the IMPACT Therapy arm of the trial will also receive supervision every 2 weeks to ensure fidelity of the intervention and to provide ongoing support and training. The supervision will be provided by research therapists by face-to-face contact, via video link or via Skype, supplemented by e-mail and/or telephone support.

Patients participating in the IMPACT therapy arm will have the option to receive three-monthly newsletters throughout the trial period (total of 4), each providing tips on healthy living. The information contained in the newsletter will comprise standard information that is readily available to the public.

### Outcome measures

Outcomes will be measured by self-report, objective assessments and face-to-face interviews. All participants will be assessed at the following time points: baseline (T0), 12 months post-randomisation (T1-at completion of intervention) and at 3 months after the end of treatment (15 months post-randomisation) (T2-) (Table 1). At each time point participants will be asked to repeat all baseline questionnaires plus provide a fasting blood sample and anthropometric measurements. 12- and 15-month follow-up windows will be defined as minus 6 weeks and plus 4 weeks for 12 month, and plus 4 weeks for 15 month follow-up. Data collected outside these time windows will be recorded but not used for the main analyses.

**Table 1:** IMPACT outcome measures at defined timepoints

**Measure**	**Baseline**	**12 months**	**15 months**
SF-36 (Physical and mental health components)	X	X	X
Time line follow back	X	X	X
Nicotine dependence questionnaire	X	X	X
AUDIT	X	X	X
DINE	X	X	X
IPAQ	X	X	X
Waist/hip circumference ratio	X	X	X
BMI	X	X	X
Blood pressure	X	X	X
Fasting glucose	X	X	X
Insulin resistance	X	X	X
Long term blood glucose control (HbA1c)	X	X	X
Lipids	X	X	X
Uric acid	X	X	X
High sensitivity C reactive protein	X	X	X
Urine drug screen	X	X	X
PANSS	X	X	X
GAF	X	X	X
MADRS	X	X	X
OPCRIT	X	X	X
LUNSERS	X	X	X
Age at randomisation	X	X	X
Gender	X	X	X
Ethnicity	X	X	X
Marital status	X	X	X
Current medications	X	X	X
Medical history	X	X	X
Measures of compliance (1–7 scale)	X	X	X

Variables measured at baseline, 12 months and 15 months follow-up.

Baseline measures:

i. Sociodemographic data; age, gender, self-reported ethnicity, marital status.

ii. Quality of life: Short Form-36; including mental health and physical health subscales [43].

iii. Substance use measures: Time Line Follow Back; [47] Nicotine Dependence Questionnaire; [48,49]Alcohol Use Disorders Identification Test (AUDIT); [50] and a urine drug screen.

iv. Biomedical status measures: waist and hip circumference; height and weight to calculate body mass index; blood pressure; fasting blood glucose; insulin (to calculate the homeostasis model assessment-estimated insulin resistance (HOMA-IR) index); long term blood glucose control (as measured by glycated haemoglobin); fasting lipids; renal and liver function tests and other markers of cardiovascular risk and inflammation.

v. Lifestyles measures: adapted Dietary Instrument for Nutrition Education DINE; [51] adapted International Physical Activity Questionnaire (IPAQ) [52].

vi. Mental health status: Positive And Negative Syndrome Scale (PANSS)[53], Global Assessment of Functioning (GAF)[54], Montgomery Asberg Depression Rating Scale (MADRS) [55].

vii. Diagnosis; OPCRIT (Operational Criteria checklist) [56].

viii. Health care: Medical history of diabetes, cardiovascular disease and cerebrovascular disease; other past medical history. Family history of diabetes and related disorders.

ix. Current medications; Composite measure of compliance scale [57]; Liverpool University Neuroleptic Side Effect Rating Scale (LUNSERS) [58].

x. Costs: adapted Client Services Receipt Inventory (CSRI) [59].

xi. IMPACT Therapy dosage measures: HPI inputs and Supervision log form.

### Sample size

The power analysis is performed for the two subscales measures, Physical and Mental health components of SF-36 Quality of Life scale.[43] We have assumed a common standard deviation of the change scores between baseline and 12 month follow-up of 10 for the Physical component score and 12 for the Mental health component score (based on the QUATRO study [60]), supplemented with further information sought directly from the study team (project reference: QLG4-CT-2001-01734, European Union)) and a cluster size of 4 patients per care coordinator with intraclass correlation of 0.05. Using 80% power, a 5% alpha level and two-tailed assumptions, a sample size of 56 care coordinators and 216 patients are needed to detect a clinical significant difference of 5 points in Mental Health Component change score (Cohen’s d = 0.42), between two groups and a sample size of 38 care coordinators and 152 patients are needed to detect a clinical significant difference of 5 points in Physical Component change score (Cohen’s d = 0.5) between two groups. Assuming a drop-out rate of 20% of the care-coordinators and their patients and an additional patient drop-out rate of 30%, a total sample size of 98 care coordinators and 392 participants are needed for the Mental health score and 70 care coordinators and 280 participants for the Physical health score.

### Data analysis

The analysis will follow the guidelines of the Consort statement for clustered randomized trials [45] and recommendations for the analysis of clustered randomized trials when presenting and analysing the data.[61,62] The trial statistician will remain blind until the main analyses are complete.

Baseline characteristics of the study population will be summarised separately within each randomised group. Baseline characteristics will also be presented for drop-outs and completers within each treatment group.

### Intention-to-treat sample

The analysis will be performed on the basis of the intention-to-treat principle and will utilise all available follow-up data from all randomised participants. Follow-up data will be collected for all patients whose care-coordinator was randomized independent of whether a care coordinator dropped out or not.

The main statistical analyses will be targeted at estimating the difference in the mean outcomes of Physical and Mental health components of SF36 [43] at 12 and 15 months follow-up observation time point between treatment and control group using a mixed effects model. [63]The outcome variables are assumed to arise from normal distributions. This will be checked and if found to be lacking then appropriate transformations will be applied.

In the linear mixed effects model Physical and Mental health Component scores respectively, at 12 months and 15 months constitute the dependent variables. “Treatment randomisation group, “time (with two levels, 12 and 15 months post-randomization)”, the interaction between “treatment group and time”, “Borough”, and the “baseline values of Physical and Mental Health scores” are the fixed part of the model. “Care coordinator” will be included as a random factor in the model to account for the dependency of the observations within a cluster. “Time” will be entered as a categorical variable.

It is possible that the treatment effects will vary across the care coordinators delivering the interventions. We will therefore also assess an “intervention group x care coordinator” interaction term. A likelihood ratio test of the corresponding variance component and information criteria comparisons will be used to assess if the interactions should be included in the model. Similarly, the interaction between treatment and time will be assessed. Should there be evidence for such an interaction then the relevant terms will be kept in the model. For the final model, the group difference estimates and associated confidence intervals will be reported for 12 and 15 months after randomization.

No *a priori* subgroups were defined in the protocol.

### Sensitivity of results to missing data

We expect that for a proportion of patients it will not be possible to measure outcome scores at 12 and/ or 15 months and that some data will be missing. The described mixed model will be fitted using maximum likelihood methods that are valid under the missing at random (MAR) assumption.[64] However, this assumption relates to the variables that are included in the model. To allow for a variable predicting “missingness”, this variable needs to be included as either one of the explanatory or dependent variables of the mixed model. We will perform three sensitivity analyses for violations of the assumptions of MAR to assess the sensitivity of the results to missing outcome data:

In the first sensitivity analysis we will use the method of multiple imputations.[64] Data handled using multiple imputation will be imputed 100 or more times, applying a set seed using the ice package in STATA version 11.0 [65]. In this procedure missing data is filled in using other information which has been observed on patients. For our analysis, the imputation model will include all variables which we believe may contain information about the missingness mechanism at 12 and 15 months and must include all variables that will be used in the main analysis model [66]. We account for clustering by including cluster indicators in our imputation model [67]. Each of these completed datasets can then be analysed using the proposed statistical modelling and the estimates from the linear mixed model will be drawn from the average of analysis of each of the completed datasets using Rubin’s Rule [64].

Analysis of data where the outcome is incomplete always requires the untestable assumptions about the missing data that they are missing at random. We, therefore, will perform a second sensitivity analyses to explore the effect of departures (varied over a plausible range) from the assumption of missing at random made in the main analysis as recommended by White et al [68]. For example, to assess for differential drop-out in patients between treatment groups a sensitivity analysis would assume that the cases lost to follow-up have systematically worse outcome than completers and outcome will be worse in treatment group and we will assess the effect of different values on the treatment differences at 12 months.

We also anticipate drop-out of care-coordinators. In a further sensitivity analysis we will perform the same analysis as described for the Intent-to treat sample using the patients whose care coordinator did not drop out. This analysis assumes that care coordinators drop-out is completely at random (MCAR) to obtain unbiased results. We will analyse the reasons for drop-out of care-coordinator and participants using a qualitative approach which allows us to assess the validity of this assumption.

### Complier average causal effects (CACE)

In addition to the standard intention-to-treat analysis we will estimate a measure of the treatment impact only for compliers. Specifically, we will employ complier average causal effects (CACE), where randomisation indicator is used as an instrumental variable to evaluate the causal effect of HPI in the subpopulations that are considered compliers to treatment. [69]This complier average causal effect is of scientific and policy interest, because it assesses the intervention effectiveness of the treatment when it is in fact taken (treatment efficacy).

### Economic evaluation

The economic evaluation will be from two perspectives: health/social care and societal (the latter additionally includes production losses due to time off work for those in employment).

To estimate HPI costs, relevant staff will document resource inputs (care coordinator training/supervision and intervention sessions with study participants). Other resource use data will be collected retrospectively by participant self-report using specifically designed interviewer-administered questionnaires at baseline, 12 months (each for the previous 6 months) and 15 months (previous 3 months). Unit costs will be attached to all resource use to estimate individual-level total costs.

Costs and outcomes will be described by arm and assessment point but the economic evaluation will focus on 15-month findings in the form of cost-effectiveness analyses based on SF-36 mental and physical component scores and cost-utility analyses based on quality-adjusted life years (QALYs). QALYs will be calculated by applying UK general population utility weights to the SF-36 [43](via the SF-6D) [70] with adjustment for relevant period of time and linear interpolation to calculate the area under the curve.

Costs and QALYs will be reported as mean values per arm with standard deviations. Differences between arms will be tested by multi-level modelling to accommodate cluster randomisation.

Given two cost perspectives and three outcome measures, there will be 6 cost-outcome combinations to consider. For combinations suggesting one arm having additional costs alongside improvements in outcomes, incremental cost-effectiveness ratios (ICERs) will represent the additional cost per additional unit of outcome. Uncertainty will be explored using incremental cost-effectiveness planes and cost-effectiveness acceptability curves (CEACs) based on the net benefit approach [71]. CEACs will represent the likelihood of the HPI being cost-effective relative to the control given different monetary values for incremental improvements in SF-36 and QALY outcomes. CEACs will be based on bootstrapped (to account for non-normally distributed data) regressions of arm upon net benefits, controlling for clusters. Sensitivity analyses will explore consequences for cost-effectiveness/utility results if key assumptions are altered (e.g. HPI unit costs, imputation for loss of follow-up).

### Process analysis

To address the research question of the process evaluation part of this trial and to try and understand the pathway from therapy to psychological change between pre-treatment and follow-up, we will carry out mediational analyses [72] using the process variables (e.g. therapist alliance) as potential mediators.

### Data management

The Clinical Trials Unit based at the Institute of Psychiatry, KCL will be employed to set up a ‘live’ database on which researchers will directly input data while assessing participants at the specified timepoints using the MACRO system.

### Ethics committee approval

Ethical approval for this study was obtained from The Joint South London and Maudsley and The Institute of Psychiatry NHS Research Ethics Committee. Ethical approval was granted on 17^th^ July 2009 (REC Ref no. 09/H080/41).

## Discussion

This paper describes the study protocol for a cluster randomised trial designed to investigate the effectiveness and cost-effectiveness of a novel non-pharmacological health promotion intervention (IMPACT therapy) in community based patients with SMI. The primary and secondary outcomes for the study are quality of life (both physical and mental health), health related behaviours, physical health parameters and change in substance misuse in a population of individuals with SMI.

There is mounting evidence to support the role of non-pharmacological interventions in improving weight gain and reducing metabolic risk in SMI. A recent meta-analysis recommended that studies of behavioural and health promotion interventions in SMI need to be of longer duration and with larger sample sizes, as well as assessing whether any effect is maintained [73]. The large sample size, long duration of follow up, and the additional 3 month follow on period to assess the maintenance of response will help bridge this gap in the evidence base. That meta-analysis also recommended measurement of a broad range of cardiometabolic risk markers and more detailed investigation of mechanisms and predictors of weight loss. Data collected as part of this RCT will allow more detailed exploration of these areas.

The trial is designed to inform real-world practice; it takes a pragmatic approach, with the intervention being delivered by the patient’s own care co-ordinator in the usual community treatment setting, yet maintains high levels of academic rigour with standardised staff training in the intervention, random allocation, and effective blinding. Provision of therapy by the usual care worker will reduce the incidence of accidental unblinding.

There remains a paucity of evidence based guidance available to guide clinicians in the treatment of physical comorbidity in this complex group of individuals with SMI. The clinical and economic results of this trial will inform service planners and clinicians alike about the potential benefits, costs and cost-effectiveness of IMPACT therapy, providing evidence as to whether national and international dissemination is justified.

## Competing interests

All authors declare that this is a National Institute of Health Research (NIHR) funded study (trial number RP-PG-0606-1049). RMM has received payment for lectures including service on speakers’ bureaus for BMS, Janssen, AZ. FG has received honoraria for advisory work and lectures from Roche, BMS, Lundbeck, and Sunovion and has a family member with professional links to Lilly and GSK; the other authors have no financial relationships with any organisations that might have an interest in the submitted work in the previous 3 years; there are no other relationships or activities that could appear to have influenced the submitted work." KI,ZA, JL, PGS , DH, DO, PL, BH, MA, AD, KG, SS have no conflict of interest or competing interests to declare. All researchers have remained independent from the funders in the completion and submission of this work.

## Pre-publication history

The pre-publication history for this paper can be accessed here:

http://www.biomedcentral.com/1471-244X/13/263/prepub

## Dissemination

Research findings will be disseminated via peer reviewed journals, conferences; internal reports, user group meetings; and the Mental Health Research Network (MHRN). When the project is completed we will be able to provide all participants with a general summary of our research through a project newsletter. Our research study is also described on the Institute of Psychiatry general website (http://www.kcl.ac.uk/iop), under the Psychosis Clinical Academic Group (CAG).
